# Anticancer Potential of Lichens’ Secondary Metabolites

**DOI:** 10.3390/biom10010087

**Published:** 2020-01-05

**Authors:** Zuzana Solárová, Alena Liskova, Marek Samec, Peter Kubatka, Dietrich Büsselberg, Peter Solár

**Affiliations:** 1 Department of Pharmacology, Faculty of Medicine, P.J. Šafárik University in Košice, 040 01 Košice, Slovakia; zuzana.solarova@upjs.sk; 2 Department of Obstetrics and Gynecology, Jessenius Faculty of Medicine, Comenius University in Bratislava, 036 01 Martin, Slovakia; liskova80@uniba.sk (A.L.); marek.samec@uniba.sk (M.S.); 3 Department of Medical Biology, Jessenius Faculty of Medicine in Martin, Comenius University in Bratislava, 036 01 Martin, Slovakia; peter.kubatka@uniba.sk; 4 Department of Experimental Carcinogenesis, Biomedical Centre Martin, Division of Oncology, Jessenius Faculty of Medicine in Martin, Comenius University in Bratislava, 036 01 Martin, Slovakia; 5 Weill Cornell Medicine in Qatar, Qatar Foundation-Education City, Doha 24144, Qatar; dib2015@qatar-med.cornell.edu; 6 Department of Medical Biology, Faculty of Medicine, P.J. Šafárik University in Košice, 040 01 Košice, Slovakia

**Keywords:** lichen, secondary metabolites, anticancer, in vitro, in vivo

## Abstract

Lichens produce different classes of phenolic compounds, including anthraquinones, xanthones, dibenzofuranes, depsides and depsidones. Many of them have revealed effective biological activities such as antioxidant, antiviral, antibiotics, antifungal, and anticancer. Although no clinical study has been conducted yet, there are number of in vitro and in vivo studies demonstrating anticancer effects of lichen metabolites. The main goal of our work was to review most recent published papers dealing with anticancer activities of secondary metabolites of lichens and point out to their perspective clinical use in cancer management.

## 1. Introduction

Despite noticeable progress in the anticancer therapy, cancer disease remains one of the major health problems worldwide [1,2]. Plant natural substances represent the rich source of active molecules that can find many applications in the field of biology, pharmacy, and medicine including oncology. Carcinogenesis is related to an imbalance between proliferation and *apoptosis*, inappropriate activity of epigenetic and transcription factors, suppression of anti-oxidative defense mechanisms and increase in angiogenesis, that leads to the consequent raising of invasiveness and formation of metastases. All these mechanisms of carcinogenesis have been well-documented as direct molecular targets for plant secondary metabolites—phytochemicals [3,4,5,6,7,8,9]. Based on many preclinical and clinical studies, phytochemicals demonstrate excellent potential how to improve the clinical state in cancer patients. The proper administration of plant natural substances may represent a significant approach how to complete the conventional management of the disease in terms of either chemoprevention or treatment. Numerous biomolecules derived from plants are also capable of synergizing with chemo- and radiotherapy. Such a combination can potentially lead to the increase of therapeutic effects and/or can reduce the side effects because lower doses of conventional therapeutics are needed [10,11,12,13,14,15].

Lichens are symbiotic organisms producing various secondary metabolites. The considerable chemical diversity of lichen secondary metabolites makes them a powerful natural source of pharmaceutical reagents that may be used in the medical practice [16]. Secondary metabolites present in lichens include aliphatic, cycloaliphatic, aromatic, and terpenic compounds, which demonstrate significant biological and pharmacological effects including those of anti-inflammatory, antiviral, antibacterial, analgesic, antipyretic, anti-proliferative, and cytotoxic [17]. These compounds have demonstrated also antineoplastic activities in preclinical research in which revealed significant effects against cancer cells [18,19,20,21,22].

The aim of this paper is to provide up-to-date knowledge about anticancer effects of lichen extracts or their secondary metabolites on different signal pathways involved in cancer and/or carcinogenesis. We discuss here the potential clinical utility of lichen secondary metabolites or natural mixtures of phytochemicals regarding the prevention and therapy of cancer. Our data were received from the English-language biomedical literature by use of “lichens” or “secondary metabolites” or “cancer” or “cell lines” and “animal models” keyword or medical subject headings (MeSH) term for searches in the PubMed bibliographic database. We have used the most recent scientific papers from the years 2015–2019.

## 2. Molecular Mechanisms of Lichen Anticancer Potential

Lichens as well as lichen secondary metabolites, also known as lichen acids, exert important abilities in the protection against carcinogenesis [23], which is due to their antioxidant, cytotoxic, pro-apoptotic, anti-proliferative, anti-migrative, anti-invasive, and overall anti-tumorigenic abilities [21,23,24,25].

Antioxidant action of most lichens is associated with the phenolic compounds, which exhibit high free radical scavenging ability [21,23]. Lichens prevent mutagenesis and/or carcinogenesis by inhibiting oxidation of cellular macromolecules [24]. Consequently, the protective effects of lichens or their metabolites against oxidative damage can be determined through the monitoring of oxidative stress markers such as superoxide dismutase (SOD) or malodialdehyde (MDA) [26].

The cytotoxic activity of lichens was observed in different cancer cell lines and we have noticed that the cytotoxic abilities of lichens in cancer cells are higher than in non-cancer cells [21,23]. First of all, the strong cytotoxic capacity of lichens against cancer cells is mediated through the processes including apoptosis, necrosis or autophagy together with the cell cycle arrest at G2/M, S, or G0/G1 phases [27]. Lichens act also as regulators of the cell cycle through the various mechanisms, such as those associated with cyclin-dependent kinases (CDK4, CDK6) or cyclin D1 [28].

In regard to programmed cell death, lichens act as activators of apoptosis in various cancer cells [21,26] through the modulation of gene expression of products related to apoptosis such as caspases, p53, p38, or anti-/pro-apoptotic proteins of Bcl-2 family [29]. Induction of apoptosis by lichens might be associated also with an increase of cleaved PARP, a stress response protein repairing damaged DNA and regulating chromatin structure [30], with inactivation of the mammalian target of rapamycin (mTOR) or activation of c-Jun N-terminal kinase (JNK) signaling [27]. Anti-proliferative effects of lichens can be modulated through the regulation of other signaling pathways such as ERK1/2 and AKT [31] or proliferation protein marker Ki-67 [32]. Interestingly, anticancer potential of lichens can also be maintained by modulation of pathways associated with the cancer invasiveness such as c-Met, which acts as mesenchymal epithelial transition factor regulating PI3K/Akt/mTOR, Paxillin/Rac-1, and STATs signaling cascades [33]. Despite regulation of STAT3 activity, [20,34] lichens target β-catenin or its downstream effectors that consequently lead to the modulation of Wnt/β-catenin target genes including the cell cycle regulating genes (c-myc, cyclin D1) as well as genes associated with cell migration (MMP7), apoptosis (BIRC5) or other regulators such as Axin2 [20,26]. Additionally, regulation of c-Jun and c-fos, members of the AP-1 family of critical regulators of gene expression and reduction of KITENIN-mediated AP-1 activity are also associated with anticancer mechanisms of lichens [20].

Indeed, anti-invasive and anti-migrative potential of lichens is related to the modulation of various signaling molecules such as members of Ras superfamily of small GTPases (RhoA, Rac1, Cdc42, and KITENIN) which play a significant role in tumor development and progression. In this regard, metastasis-related genes including CAPN1, CDC42, CFL1, IGF1, or WASF1 as well as epithelial–mesenchymal markers (Twist, Snail, Snug) might also be targets of lichens. On the other hand, anticancer effect of lichen realized through the angiogenesis inhibitory activities are related to the suppression of endothelial tube formation [35] or vascular endothelial growth factor receptor (VEGFR)-2-mediated Akt and extracellular signal-regulated kinase (ERK) signaling [29]. Current results showed that anticancer effects of lichens are also associated with the modulation of inflammatory responses via TNF-α, IL-1β, IL-6, and TGF-β1 [36] and with targeting of microRNA molecules [37].

**Figure 1:**
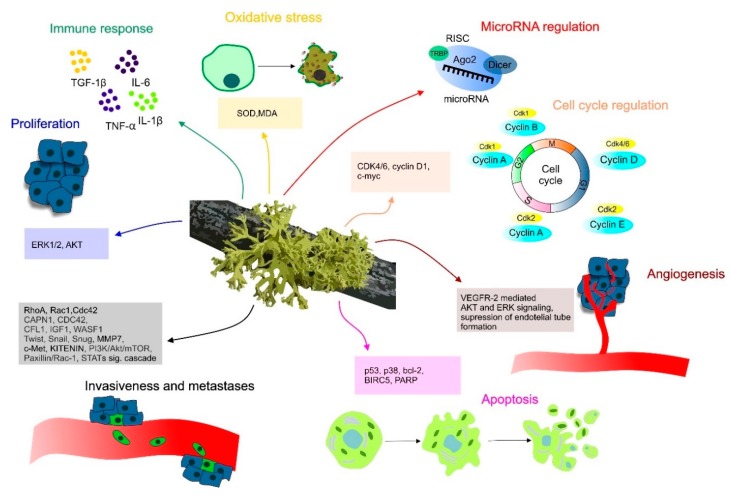
The role of lichens in the modulation of molecular mechanisms associated with cancer.

Overview of cancer-associated molecular mechanisms modulated by lichens is shown in Figure 1. Table 1 shows secondary metabolites of lichens, their origin, chemical structure and also their beneficial activities including anticancer one.

## 3. Anti-Neoplastic Effect of Lichens in Preclinical Research

### 3.1. In Vitro Evaluation of Anticancer Efficacy of Isolated Lichen Compounds

Pro-apoptotic abilities of usnic acid (UA), a secondary lichen metabolite isolated from *Usnea diffracta*, was evaluated in human colorectal adenocarcinoma (CaCo2), human rhabdomyosarcoma (RD), human cervical carcinoma (Hep2C), human hepatocellular carcinoma (HepG2), mouse fibrosarcoma (Wehi), as well as in nonmalignant African green monkey kidney (Vero) and mouse subcutaneous connective tissue (L929) cell lines. Both natural and commercial UA had similar cytotoxicity on cancer cells but no significant effect in non-malignant L929 and Vero cells. Indeed, UA increased the expression of Bax and decreased the expression of Bcl-2 and p53 genes in cancer but not in non-malignant cells [29]. Additionally, the concentration of UA at 8 μM, inhibited SCF-mediated migration of human colorectal cancer (HCT116, LS174 c-KIT+) cells. UA induced an inhibition of mTOR activity in HCT116 cells thereby activated suppression of protein kinase C alpha type (PKC-A) and induced an autophagy followed by the degradation and transcriptional inhibition of mast/stem cell growth factor receptor Kit (c-KIT) as well as to an increase in LDH release [68]. Another study of UA demonstrated its ability to inhibit the proliferation of human gastric carcinoma (BGC823, SGC7901) cells through the G0/G1 and G2/M cell cycle arrest, respectively while BGC823 cells were more sensitive. Both apoptosis and autophagy of BGC823 and SGC7901 cells with increased ratio of Bax/Bcl-2 and increased activity of caspase-3 and PARP cleavage after UA therapy were identified [69]. Anti-proliferative effects of UA were also evaluated in human breast cancer cell lines in which 67, 15, and 8 miRNAs in MDA-MB-231, BT-474, and MCF-7 cells, respectively after UA therapy were detected. Indeed, enrichment analysis revealed different groups of miRNA with targets as follows: Apoptosis, Hedgehog, TGF-β, and MAPK pathways identified as prominent [37]. Pro-apoptotic abilities of UA were demonstrated through the G0/G1 and G2/M cell cycle arrest in both hepatocellular carcinoma HepG2 and SNU-449 cells [27]. Similarly, an application of UA in lung cancer cells (A549) led to the decrease of cells, induced apoptosis and inhibited cell proliferation, which was accompanied by G0/G1 cell cycle arrest, decreased expression of CDK4, CDK6, and cyclin D1 and increased expression of CDK inhibitor (CDKI) p21/cip1 protein. Consequently, the apoptotic effect of UA co-occurred with enhanced PARP cleavage [28]. The anticancer effects of UA isolated from *Cladonia arbuscula* were demonstrated through the formation of autophagosome as a consequence of H+ shuttling in mitochondria and lysosomes in human breast cancer (MCF-7, T47D) as well as in human pancreatic cancer cell lines (Capan-2) with the decrease in ATP, activation of AMP-kinase and the detection of cellular stress markers [70]. Similarly, UA induced an apoptosis of MCF-7 cells through the generation of ROS and mitochondrial/caspase pathway. On the contrary, N-acetylcysteine blocked ROS generation, reduced apoptosis mediated by c-Jun-N-terminal kinase, caused a loss of mitochondrial membrane potential, released the cytochrome-c and activated caspases [71]. In another study, UA isolated from several lichens (*Alectoria samentosa*, *Flavocetraria nivalis*, *Alectoria ochroleuca*, and *Usnea florida*) showed the significant inhibitory potential against migration and invasion of human lung cancer (A549, H460, H1650, and H1975) cells. It was also associated with the inhibition of A549 cells motility. Anti-invasive efficacy of UA in H1650 and H1975 cells was demonstrated by the decline in β-catenin-mediated TOPFLASH and KITENIN-mediated AP-1 activity. Additionally, synthetic derivatives of UA exerted anti-proliferative activity in human prostate cancer (PC-3), human epithelial carcinoma (HeLa), and MCF-7 cells. All active derivatives arrested cells at G0/G1 phase and decreased the fraction of HeLa cells in S and G2/M phases. Derivatives 2a and 2b arrested also MCF-7 cells at G0/G1 phase and induced the apoptosis of these cells. Derivatives 2a and 2b also activated strong cytoplasmic vacuolization, which was associated with dynein-dependent endocytosis. This mechanism has not yet been demonstrated in UA and it is the first time reported in synthetic derivatives of UA [72]. The potasium usnate (PU), which was prepared to enhance the solubility of UA, demonstrated cytotoxic activity in each tested colorectal cancer cells (human HCT116, DLD1, SW480, HT29, SW620, Caco2, COLO320, and mouse CT26) with lower IC50 values than UA except of SW480 and CT26 cells. Potasium usnate showed stronger inhibitory effects on the invasion of Caco2 and HCT116 cells compared to UA. Importantly, PU downregulated the epithelial–mesenchymal markers (EMT) including Twist, Snail, and Snug and decreased the expression of metastasis-related genes CAPN1, CDC42, CFL1, IGF1, WASF1, and WASL in Caco2 cells [34]. An anticancer efficacy of UA was evaluated together with diffractaic acid (DA) and lobaric acid (LA) in human glioblastoma multiforme (U87MG-GBM) and rat cerebral cortex cells (PRCC). Lobaric acid exerted highly toxic effects demonstrated by the drop of cell viability to 35.09% in PRCC and 30.47% in GBM cells, while DA and UA exerted greater total antioxidant capacity (37.74 and 37.34 mmol Trolox equivalent/L, respectively) in PRCC cells when compared with other compounds [73]. Cancer inhibitory effect of UA and atranorin (ATR) was also evaluated in human melanoma (HTB-140) and human prostate cancer (DU-145, PC-3) cell lines. Interestingly, both UA and ATR inhibited cancer cells proliferation, migration and actin organization while their effects on apoptosis were less significant [74]. An impact of UA isolated from *Cladonia foliacea* Wild and vulpinic acid (VA) isolated from *Letharia vulpina* Hue on proliferation and viability was evaluated in HepG2, mouse neuroblastoma NS2OY and human umbilical vein endothelial (HUVEC) cells. Although UA was more cytotoxic against all cell lines, it had higher anti-proliferative effects in HepG2 cells. On the other hand, VA inhibited the proliferation of NS2OY cells more effectively. Interestingly, the cytotoxic effects of both metabolites against HUVEC were only mild. Moreover, both UA as well as VA exhibited anti-angiogenic abilities evaluated by the endothelial tube formation assay [75]. Vulpinic acid also decreased viability and induced apoptosis of human breast cancer cells (MCF-7, MDA-MB-231, BT-474, SK-BR-3) compared to human non-malignant breast epithelial cells (MCF-12A). An evaluation of apoptosis-related genes showed that the expression of p53 after VA therapy was almost six times higher in SK-BR-3 cells than in MCF-12A cells [47]. Similarly, an apoptotic activity of VA was evaluated in vitro in CaCo2, HepG2, Hep2C, RD, Wehi as well as in normal Vero and mouse L929 cells. Vulpinic acid inhibited growth of all tested cell lines in a time and dose-dependent manner and a higher efficacy was found in CaCo2 cells. Vulpinic acid also exhibited significant cytotoxic effects on all tested cancer cells. On the other hand, it did not exert any significant cytotoxicity of on normal L929 and Vero cells, but interestingly, all mRNA, Bax protein levels and p53 were more significantly increased in cancer compared to normal cells. In addition, mRNA and Bcl-2 protein levels showed 7 fold decrease in HepG2 and CaCo2 cells and 5–6 fold decrease in Hep2C, RD and Wehi cells [76].

Similarly, natural compound ATR, isolated from lichens, was tested against mouse breast cancer (4T1) cells. ATR reduced the clonogenic potential of 4T1 cells compared to normal mammal non-malignant epithelial (NMuMG) cells, in which the clonogenic ability remained unaffected. BrdU incorporation assay did not confirm the anti-proliferative effect of ATR in 4T1 cells. On the contrary, ATR induced caspase-3 activity, PARP cleavage and depletion of Bcl-xL in 4T1, but not in NmuMG cells [45]. Atranorin, isolated from *Stereocaulon caespitosum* also inhibited the growth of human hepatocellular carcinoma (SK-Hep1, Huh-7, SNU-182) cell lines when used in concentration higher than 10 μg/mL. Atranorin arrested SK-Hep1 cells at G2/M phase, induced cell death at 24 h time point and suppressed migration and invasiveness of Sk-Hep1 and Huh-7 cells [77].

However, only high concentrations of ATR and gyrophoric acid (GA) had similar effect on human melanoma A375 cells, physodic acid (PA) induced apoptosis in A375 cells by mechanism probably involving the downregulation of HSP70 [17]. In this regard, Emsen et al. analyzed the effect of PA together with olivetoric acid (OA) and psoromic acid (PSA) on U87MG and rat PRCC cells and found a positive correlation between the cytotoxicity associated with the three tested metabolites and their concentrations, lactate dehydrogenase (LDH) activity, and oxidative damage of DNA [43].

Furthermore, *Parmotrema dilatatum*, *Parmotrema lichexanthonicum*, *Usnea subcavata*, *Ramalina sp.*, *Dirinaria aspera*, *Cladina confusa* and their secondary metabolites were tested on melanoma cancer (UACC-62), murine melanoma (B16-10), and human fibroblast (NIH/3T3) cells. Protocetraric acid (PrA), norstictic (NA), and PSA (depsidones) acids together with divaricatic (DiA) and perlatolic (PeA) (depsides) acids showed a strong cytotoxic effect on UACC-62 cells and reached higher selectivity for melanoma cells compared to 3T3 normal cells. In this regard, NA and DiA was also the most effective against B16-F10. Protocetraric acid proved to be the best candidate for in vivo studies of melanoma since it showed the highest selectivity index against UACC-62 cells [78].

Paluszczak et al. evaluated effects of lichen-derived compounds on Wnt signaling in colorectal cancer (HCT116, DLD-1) and immortalized keratinocyte (HaCaT) cell lines. Caperatic acid (CA) isolated from *Platismatia glauca* downregulated β-catenin-regulated expression of Axin2 gene in both colorectal cancer cell lines, but lecanoric acid (LeA), obtained from *Hypocenomyce scalaris* decreased the expression of Axin2 in HCT116 cells just moderately. On the contrary, CA and PA (isolated from *Hypogymnia physodes*), downregulated the expression of MMP7 and survivin in HaCat cells in a concentration-dependent manner. Furthermore, CA inhibited the migration of both colorectal carcinomas by 20%, while PA did not have any effect on the cell migration. Caperatic acid showed also the strongest cytotoxic effects on cancer cells at the concentration of 100 μM while other metabolites in this concentration demonstrated just moderate efficacy [26].

Barbatic acid (BA), isolated from acetone extract of *Usnea longissima*, exhibited strong cytotoxic activity against HeLa, A549, MCF-7, and DU-145 cells. Barbatic acid arrested A549 cells in G0/G1 phase with 71% of cell accumulation at the concentration of 1 μM and the same concentration induced the apoptosis of A549 cells with increased caspase-3 activity, PARP cleavage, annexin V staining, and chromatin condensation [38].

In addition, anti-angiogenic and anti-migratory efficacy of lichen-derived small molecule barbatolic acid (BrA) isolated from acetone extract of *Bryoria capillaris* was evaluated on T-47D, HUVEC, and cisplatin-resistant BRCA2-mutated human breast TNM stage IV adenocarcinoma cells (HCC1428). Sub-cytotoxic concentrations (25–100 µM) of BrA dose-dependently inhibited both endothelial tube formation as well as migration determined by the scratch wound healing assay. In fact, the effect of BrA on the migration of cancer T-47D and HCC1428 cells was more effective than on normal HUVEC cells [35].

Hypostictic acid (HA), extracted from *Pseudoparmelia sphaerospora*, exerted significant anti-proliferative efficacy against chronic myelogenous leucaemia (K562), murine melanoma (B16-F10), and renal cancer (786-0) cell lines. Salazanic acid (SA) obtained from *Parmotrema cetratum*, showed in vitro anti-proliferative effects also in K562 and B16-F10 as well as in colon cancer (HT-29) cell lines [79]. In this regard, SA and protocetraric acid (PrA) are major phenolic compounds in lichens including *Parmelia caperata*, *Parmelia saxatilis* and *Parmelia sulcate*. Manojlovic et al. evaluated an impact of the above-mentioned compounds against human melanoma FemX and LS174 cell lines [80].

Retigeric acid B (RA-B), a pentacyclic triterpenic acid isolated from *Lobaria kurokawae* induced apoptosis in PC-3 and DU145 cells through the inhibition of IκBα and p65 (subunit of NF-κB) phosphorylations. In addition, microarray analysis revealed alterations in the expression of genes associated with cellular processes including apoptosis, invasion, and proliferation after RA-B application [81].

Protolichesterinic acid (PLA) isolated from *Centraria islandica* exerted anticancer efficiacy in SK-BR-3 and T47D cell lines while anti-proliferative effects were also observed in SK-BR-3 cells. Indeed, this metabolite increased the expression of fatty acid synthase and decreased the expression of HER2 in SK-BR-3 cells. Additionally, downregulation of ERK1/2 and AKT signaling was observed probably due to reduced HER2. There was also demonstrated some synergistic effect of PLA and lapatinib in SK-BR-3 cells [31]. Similarly, PLA affected HeLa, K562, and human neuroblastoma cell lines (SH-SY5Y). Interestingly, PLA combined with doxorubicin exerted synergistic cytotoxicicity in HeLa cells but not on SH-SY5Y and K562 cells. The mechanism of synergistic effects might be associated with the apoptosis induced by both PLA as well as doxorubicin, which can induce caspases -3, -8, and -9 activities. Bim expression, which mediates cytochrome-c release might be increased in an additive manner by both PLA and doxorubicin. Protolichesterinic acid also seems to behave as a competitive inhibitor of fatty acid synthase [82]. Bessadóttir et al. also tested effects of PLA (from *Cetraria islandica*) in human multiple myeloma (RPMI 8226), (U266) and human pancreatic carcinoma (AsPC-1). Moreover, PLA inhibited the proliferation of RPMI 8226, U266, and AsPC-1 cells with very low IC_50_ values. It induced the cell cycle arrest of pancreatic AsPC-1 cells in G1 phase and the apoptosis of RPMI 8226 and U266 cells but not the apoptosis of pancreatic AsPC-1 cells. Indeed, the pro-apoptotic activity of PLA was cancer cell line-dependent while high concentrations of PLA inhibited the production of 5- and 12-HETE but only in pancreatic not in myeloma cells [83].

Ramalin (RAM) a metabolite isolated from *Ramalina terebrata* inhibited at high concentrations (50 and 100 μg/mL) the proliferation of HCT116 cells. Ramalin induced G2/M cell cycle arrest through the upregulation of TP53 and p21 and downregulation of cyclin B1 and CDK1. The highest concentration of RAM (100 μg/mL) induced significant number of apoptotic cells. Furthermore, wound healing, invasion, and migration of HCT116 cells were also dose-dependently suppressed after the treatment with RAM [84].

Physciosporin (PHY) isolated from *Pseudocyphellaria granulata*, was evaluated in colorectal cancer cell lines (CT26, Caco2, HCT116, DLD1, SW620). While toxic concentrations of PHY induced apoptosis of tested cancer cells, non-toxic ones inhibited migration, invasion, and colony formation of colon cancer cells in concentration-dependent manner. Physciosporin down-regulated downstream transcription factors and/or target genes of EMT, KITENIN, β-catenin and reduced actin-based cell motility [22]. Another PHY, isolated from *Pseudocyphellaria coriacea* demonstrated anticancer effects in A549, H1650, and H1975 through inhibition of migration and invasion of human lung cancer cells through the downregulation of N-cadherin, KITENIN-mediated AP-1 activity, Cdc42, and Rac1. Interestingly, the metastasis suppressor gene KAI1 was also upregulated by PHY [85].

Interestingly metabolites isolated from acetone extract of *Dirinaria consimilis*: antarvediside A (antarA), antarvediside B (antarB), sekikaic acid (SeA), ATR, divaricatic acid (DiA), 2′-O-methyl DiA revealed anticancer potential. In this regard, antarB at the concentration of 30 μg/mL inhibited the growth of HeLa and MCF-7 stronger than the doxorubicin at the concentration of 10 μg/mL. Only metabolites antarB and 2′-O-methyl DiA inhibited the proliferation of A549 cells. Importantly, all metabolites had lower toxicity against human mammary epithelial normal NHME cells in comparison to cancer cells [86]. Table 2 shows an overview of anticancer effects of above-mentioned isolated lichen secondary metabolites.

### 3.2. Combined Studies of Lichen Extracts and Isolated Lichen Compounds In Vitro

Notably, several authors studied anticancer potential of lichen extracts in combination with isolated lichen compounds. An acetone extract of *Flavocetraria cucullata* or its subcomponent UA exerted a selective cytotoxicity on cancer cells by inducing apoptosis at lethal concentrations. Sub-lethal concentrations of this extract and UA inhibited tumorigenesis and motility of cancer cells, suppressed epithelial–mesenchymal transition (EMT) and inhibited Akt phosphorylation. Interestingly, the anticancer activity of the extract was more potent than that of UA [87].

Another study analyzed anticancer properties of acetone extract of *Toninia candida* and *Usnea barbata* as well as their major compounds nortictic acid (NA) and UA against FemX and LS174 cells using tetrazolium assay. The results of the study proved the anticancer activity of NA, UA, and lichen extracts in both cell lines. Importantly, UA demonstrated the strongest cytotoxic effect while both NA and UA induced apoptosis in FemX and LS174 cells [54]. Moreover, anticancer and antioxidant availabilities of supercritical CO_2_ extract of old man’s beard (*Usnea barbata*) (SCE) compared to extracts obtained through conventional methods (Soxhlet extracts and macerate) were evaluated in mouse melanoma (B16), rat glioma (C6), and HaCaT cells. SCE exerted the highest cytotoxic effect on both B16 and C6 cells. The cytotoxic effect of lichen extracts correlated well with the content of UA (the most abundant compound) and ROS production. The cytotoxic effects of extracts were evaluated for apoptosis and autophagy processes via monitoring of cells for cell cycle phases and formation of acidic cytoplasmic vesicles. Consequently, both, SCE and UA induced apoptosis and/or autophagy in B16 and C6 cells. Indeed only very low toxicities of all tested extracts were demonstrated against normal HaCaT cells [88].

Furthermore, acetone extracts of lichens including *Evernia prunastri* and *Pseudoevernia furfuraceae* and their major secondary metabolites, mainly PA, demonstrated anticancer activity in FemX and LS174 cells using MTT test. Physodic acid exerted the best cytotoxic effect against both cell lines. Additionally, authors also observed a reduction of FemX and LS174 cells in the S and G2/M phase. On the other hand, there was an increase in sub-G1 population of cells after the treatment with mentioned above extracts and metabolites [89]. Similarly, PA (isolated from *Hypogymnia physodes*) demonstrated the most significant cytotoxic effect on MCF-7 cells followed by T47D and MDA-MB-231 cells. The viability of MCF-10A cells was not changed even at concentration of PA higher than 100 μM [24].

Ethanol extract of *Usnea strigosa* as well as its compound NA also showed anticancer effects in MDA-MB-231, MDA-MB-468, MCF-7, T-47D, BT-474, and SK-BR-3 cells. NA exerted anti-proliferative effects in all six breast cancer cell lines with the best inhibition demonstrated in MDA-MB-231 and MDA-MB-468 cells. Nortictic acid inhibited the migration of MDA-MB-468 cells and invasion of MDA-MB-231 cells through the basement membrane. On the other hand, they did not show any significant cytotoxic effects on human non-tumorigenic MCF-10A cells. This acid suppressed also c-Met, STAT3, paxillin/Rac-1and FAK phosphorylation in MDA-MB-231 cells [33]. Moreover, acetone extract of *Melanelia subaurifera* and *Melanelia fuliginosa* and their compounds LeA and 2′-O-methyl anziaic acid (2′-O-MA) suppressed HeLa, A549, and LS174 cells. Indeed, LeA and 2′-O-MA metabolites had lower cytotoxic effects compared to both extracts, while both extracts as well as metabolites did not exerted any cytotoxicity on normal MRC5 cells [25].

Ether extract of *Cladonia salzmannii* showed cytotoxic effects on human acute promyelocytic leukemia (HL-60), human laryngeal carcinoma (HEP-2), human lung mucoepidermoid carcinoma (NCI-H292), and murine macrophage (RAW-264.7) cells. In this regard, acetone extract revealed cytotoxic effects on MCF-7 and NCI-H292 cells while purified BA exerted cytotoxicity on HEP-2, MCF-7 and RAW-264.7 cells [90]. Barbatic acid a secondary metabolite of *Cladia aggregate*, was applied against HEp-2, human squamous cell lung cancer (NCI-H292), and human nasopharyngeal squamous cell carcinoma (KB). Lichen extract as well as the isolated compound showed the cytotoxicity against all tested cancer cell lines while the most sensitive ones were HE-p2 cells [91].

Acetone extract of *Everniastrum vexans* inhibited the migration of A549 cells (10 μg/mL). Atranorin identified as an active secondary metabolite of the extract showed the cytotoxic effect on A549 cells at concentrations higher than 5 μg/mL. Atranorin inhibited TOPFLASH activity mediated by β-catenin reducing nuclear import of β-catenin and suppressing the expression of c-jun/AP-1 target genes. Moreover, ATR decreased the mRNA expression of KITENIN and increased the expression of KAI1 mRNA. It also decreased the level of GTP-Cdc42, GTP-RhoA and STAT proteins [20].

In addition, acetone extract of the endolichenic fungus EL002332 isolated from *Endocarpon pusillum* exerted selective cytotoxicity on human gastric cancer AGS and mouse colon cancer CT26 cell lines. It also showed synergistic effects on human gastric cell lines (AGS, TMK-1) in combination with docetaxel chemotherapy. Active compound isolated from acetone extract (myC) had even higher anticancer effect on AGS cells than the crude extract. In fact, myC induced apoptosis through the activation of caspase activity and regulating the expression of Bcl-2 family proteins [92].

Table 3 summarizes combined studies of lichen extracts and isolated lichen compounds evaluating their anticancer effectiveness.

### 3.3. Determination of Lichens’ Extracts Effect Against Cancer Cells

In addition, beneficial anticancer activity is associated also with lichen extracts. As revealed by Kosanic et al., extracts of *Umbilicaria crustulosa*, *Umbilicaria cylindrica*, and *Umbilicaria polyphylla* showed strong anti-neoplastic effects against FemX and LS174 cell lines evaluated using MTT assay [93]. Similarly, methanol extract of *Lasallia pustulata* and extracts from *Parmelia caperata*, *Parmelia sulcate*, and *Parmelia saxatilis* showed strong cytotoxic activity against FemX and LS174 [94,95]. Another study evaluated the anticancer effect of 70% methanolic extract of *Parmotrema reticulatum* against MCF-7 and A549 cancer cells and normal fibroblast WI-38 cells. Analyzed data documented the strong cytotoxic activity of this extract on MCF-7 cells, while low cytotoxicity on A549 and WI-38 cells. Moreover, the extract induced S and G2 cell cycle arrest of MCF-7 cells associated with decreased expression of B1, Cdk2, Cdc25C, and increased expression of p53 and p21. An increased ratio of Bax/Bcl-2 and higher caspase activity were detected in MCF-7 cells leading to PARP cleavage and apoptosis [96]. Another study demonstrated anticancer activity of methanol extract of *Cetraria islandica* on FemX and LS174 cells [97]. In addition, Kosanic et al. studied biological activity of *Parmelia arseneana* and *Acarospora fuscata* acetone extracts against FemX, LS174, A549, and human chronic myeologenous leucaemia cells K562. Their results revealed strong anticancer effects of the *P. Arseneana* extract in all tested cell lines [98]. All extracts of unique trans-Himalayan lichens demonstrated a cytotoxic effect against HepG2 and human colon cancer (RKO) cells. Moreover, methanol extract of *Lobothallia alphoplaca* and *Melanelia disjuncta* exhibited high cytotoxicity against both cancer cell lines [99]. Additionally, treatment of A549, PC-3, Hep3B, and C6 cell lines with methanol extracts of *Parmelia sulcata Taylor* and *Usnea filipendula Stirt* led to the cytotoxic, genotoxic and apoptosis-inducing effects in all tested cell lines [100]. Another in vitro experiment of methanol extract of *Hypogymnia physodes* (low concentrations) indicated anticancer and/or apoptosis-inducing effects on MCF-7 and MDA-MB-231 cells and the genotoxic (high concentrations) impact on human lymphocytes [101]. Moreover, methanol extracts of *Cladonia rangiformis* and *Cladonia convolute* exerted a significant anti-proliferative effect and these extracts induced also significant apoptosis of MCF-7 cells demonstrated by TUNEL assay [102]. Additionally, acetone extract of *Xanthoria parietina* represents a unique mixture of the secondary metabolites with anticancer abilities. *X. parietina* extract inhibited the proliferation of MCF-7 and MDA-MB-231 cells with the maximum effect observed at the concentration of 1.5–3 mg/mL. This extract induced the G1 cell cycle inhibition with the upregulation of p27 and p16 and downregulation of cyclin D1 and cyclin A. Interestingly, therapy of MCF-7 and MDA-MB-231 cells with *X. parietina* decreased the expression of anti-apoptotic Bcl-2 and increased the expression of pro-apoptotic TRAIL and pBAD proteins [103]. In addition, a metanol extract of *Parmelia sulcata* had a cytotoxic effect on both MCF-7 and MDA-MB-231 cells. The apoptosis induced in MCF-7 and MDA-MB-231 cells by *P. sulcata* extract was rather caspase-independent due to the lack of changes in caspase-3 activation or in PARP cleavage [104]. Recently, researchers evaluated crude extracts of 17 lichen species against Human Burkitt’s lymphoma (Raji). *Xanthoparmelia chlorochroa* and *Tuckermannopsis ciliaris* extracts induced apoptosis (dose-dependent), accumulation of cells in the GO/G1 stage and increase of p53 protein [105]. Acetone extracts of *Parmotrema gardneri*, *Pannaria sp.*, and *Canoparmelia aptata* applied to human gastric adenocarcinoma (AGS) and A549 cancer cells as well as normal Canine Madin–Darby kidney cells (MDCK) exerted anticancer potential. On the contrary, *C. aptata* extract had just weak cytotoxicity on AGS and A549 cells [106]. Additionally, *Cladonia rangiformis* and *Cladonia convolute* represent fruticose lichens with antimicrobial and cytotoxic effects. The extracts from both lichens showed strong cytotoxic impact on MCF-7 cells [107].

Furthermore, *Caloplaca pusilla* grown on G-LBM medium decreased cell viability and induced apoptosis of MCF-7, PC-3 and HeLa cells in concentration-dependent manner. Mycelia of *Xanthoria parietina* grown on PDA and G-LBM decreased cell the viability of MCF-7 and HeLa cell lines [108]. Also, methanol extract of *Cladonia pocillum* increased the apoptosis in MCF-7 cells in concentration-dependent manner [109]. Extract of *Pleurosticta acetabulum* exerted strong cytotoxic effect on HT-29 cells. This extract also inhibited the proliferation of HT-29 cells through the presence of cytochalasin E and induced apoptosis [110]. Furthermore, polysaccharide from *Umbilicaria esculenta* decreased the viability of human melanoma A875 and A375 cells but did not exhibit any cytotoxic effect on HUVEC cells. Both, Annexin-V positive as well as TUNEL positive A875 cells were induced after this therapy in concentration-dependent manner. In this regard, apoptosis of A875 but not HUVEC cells was induced by polysaccharide of *U. esculenta* (as a result of ROS generation) followed by increased expression of caspase-3 and -9 [111]. An evaluation of biological activities of five lichen species of Cladonia genus revealed the highest cytotoxic effect of *Cladonia foliacea* extract on A549 and human colon cancer (LS174) cells. Moreover, the highest cytotoxic effect on HeLa cells was observed after the treatment with the extract of *Cladonia furcata* [112]. In addition, high concentration of *Candelariella vitellina* extract reduced the proliferation (Ki-67) and induced apoptosis and necrosis of Caco-2 cells. The extract of *C. vitelline* decreased Bcl-2 but increased Bax and CASP3 protein levels, so the ratio of Bax/Bcl-2 increased [32]. Anticancer potential of methanol extracts of lichens was evaluated in human lung (H1299, A549) and breast (MDA-MB-231, MCF-7) cancer cell lines. The extract of *Usnea intermedia* exerted the strongest anti-proliferative effects especially observed in H1299 and MDA-MB-231 cells. The extract of *U. intermedia* also induced apoptosis confirmed via the phosphatidylserine translocation, increased caspase 3/7 activity, loss of mitochondrial membrane potential and the formation of pyknotic nuclei [113]. Isolates from *Nemania serpens* and *Nemania aenea var. aureolatum* were the most active compounds against human colorectal cancer (HT-29, HCT116) and human prostate cancer (PC-3, DU145) cell lines. These isolates induced apoptosis characterized by activated caspase 3, 8, PARP cleavage and chromatin fragmentation [114]. In addition, Nugraha et al. evaluated anticancer activities of nine lichens of East Java Indonesia, of which *Physcia* cf. *Milegrana* showed the most significant cytoxicity, but only on HeLa cells. Other metabolites exhibited very low cytotoxicity either in cancer or normal African green monkey kidney (Vero) cells [115]. Table 4 shows an overview of anticancer effects of above-mentioned lichen extracts in various cancer cell lines.

## 4. Anticancer Effects of Lichens in Animal Models

Anticancer activities of naturally occurring plant compounds and extracts derived from lichens are supported by numerous studies using animal xenograft/allograft models. There are several studies evaluating the anticancer potential of UA in vivo. Usnic acid inhibited angiogenesis in both chick embryo chorioallantoic membrane as well as in VEGF-induced mouse corneal angiogenesis model. Moreover, UA inhibited the growth of human Bcap-37 BC cells inoculated into C57BL/6 female nude mice and suppressed the angiogenesis in tumor tissue. The inhibition of angiogenesis was evaluated by anti-CD31 and showed the reduction in integrated optical density of tumor blood vessels in the UA-treatment group. In this regard, in vivo study was supported by the parallel in vitro evaluation, where UA reduced proliferation, migration, and tube formation of HUVEC cells, blocked VEGFR2 mediated ERK1/2 and AKT signaling and induced apoptosis via decrease in Bcl-x1 and survivin levels and through the elevation of caspase 3 activity and PARP cleavage [116]. Furthermore, UA inhibited tumor growth of human MCF-7 breast cancer-bearing mice dose-dependently through the generation of ROS and JNK stimulation associated with mitochondrial/caspase pathway leading to apoptosis of cancer cells. Indeed, UA was well tolerated and did not show any toxic effects in animals [71]. This metabolite reduced also toxicity of bleomycin therapy in Kunming mice with inoculated mouse H22 hepatocellular carcinoma cells and the combination of these molecules was more effective against H22 cancer compared to single bleomycine. Indeed, the combination of UA and bleomycin arrested tumor cells at G0/G1 phase and induced the apoptosis via caspase-3 and -8 activation (cleavage) probably as a consequence of transcription regulation of p53/p21/cyclin pathway. Furthermore, UA decreased the level of MDA, hydroxyproline, TNF-α, IL-1β, IL-6, and TGF-β1 and its combination with bleomycin increased the level of SOD in lung tissues of H22-bearing mice probably through the downregulation of p-Smad2/3 and the upregulation of Smad7 proteins [36]. Furthermore, benzylidene analogue of UA demonstrated anticancer activity in vivo on two MDA-MB-231 and MCF-7 xenograft mouse breast cancer models. Both UA as well as its benzylidene analogue induced autophagy and the inhibition of mTOR signaling pathway accompanied by significantly decreased level of mTOR downstream effectors p-S6K and p-4E-BP1 in treatment groups of both models [117]. In the mouse model of human gastric BGC823 carcinoma, UA decreased the volume and weight of tumors without any weight loss of animals. Moreover, evaluating the Bax/Bcl-2 ratio, UA exhibited stronger pro-apoptotic activity, when compared to 5-FU. Although 5-FU had the same effect on tumor volume and weight, it was associated with significant animal weight loss [69]. In another experiment, Nguyen et al. [87] tested anticancer activities of *Flavocetraria cucullata acetone extract*, UA, and LiA on human A549 lung cancer cells using xenograft Balb/c mouse model [87]. The highest tumor free survival number was found in *F. cucullata* pretreated group (tumor free in six out of eight mouse) compared to DMSO group (zero out of eight), UA group (four out of eight) or LiA group (two out of eight) [87].

Interestingly, PU demonstrated better bioavailability in the tumor, liver, and plasma compared to UA in CT26-Fluc syngeneic mouse tumor model. Potassium usnate inhibited the growth of colorectal cancer cells and inhibited liver metastasis probably through the reduction of EMT markers such Twist, Snail, and Slug and the metastasis-related genes CAPN1, CDC42, CFL1, IGF1, WASF1, and WASL in this model. In addition, PU did not have any hepatotoxic effect in the mouse liver metastasis model [34]. Interestingly, *ethyl acetate extract of Usnea longissima prevented esophagogastric adenocarcinoma* induced by oral administration of N-methyl-N-nitro-N-nitrosoguanidin *in* Albino Wistar male rats. *U. longissima extract* demonstrated prominent anticancer effect and selectivity to cancer tissue in animals at concentrations of 50 and 100 mg/kg without any toxic effects. This extract did not reveal any lethal effect even when administered in high concentrations (500, 1000, and 2000 mg/kg) [118].

Study from our laboratory showed significant anticancer effects of ATR in mouse 4T1 breast cancer allograft model in BALB/c mice. Atranorin significantly increased survival time of tumor-bearing animals, reduced the tumor volume and had rather direct proapoptotic than anti-proliferative effect on tumor cells. In addition, ATR protected livers of tumor-bearing mice against oxidative stress [45]. In another study, ATR reduced tumor volume and weight and diminished Ki-67 marker of proliferation in Lewis lung carcinoma xenograft model in C57BL/6 mice. Moreover, ATR decreased the expression of KITENIN, CD44, STAT, and cyclin-D1 genes in both in vitro as well as in in vivo conditions [20].

There are also several more lichen extracts or metabolites that were evaluated in cancer animal models. In this regard, Poornima et al. evaluated the extract of *Rocella montagnei* against Dalton’s lymphoma ascites cells, that were inoculated into Albino Wistar rats and resulted in suppression of tumor growth. Briefly about experiment workflow: Thirteen days after inoculation, cancer fluid was aspirated from the peritoneal cavity of rats and consequently injected into another group of animals. Analyzed data demonstrated, that lichen extract reduced the volume of tumors and the effect was comparable to standard therapy with Vincristine [119]. In another study, the anticancer effects of *endolichenic fungus EL002332* (*Endocarpon pusillum*) extract was evaluated in mouse colorectal CT26 cancer cells that were inoculated into BALB/c syngeneic mice. TMK1 cells were injected into the abdominal cavity of BALB/c mice to establish intraperitoneal xenografts. Both tumor score and tumor volume were significantly reduced in skin and intraperitoneal tumor-bearing animals after EL002332 crude extract [92]. El-Garawani et al. tested anticancer activities of *Candelariella vitelline* extract on Ehrlich ascites carcinoma cells that were injected and consequently transferred every 5 days to new Swiss albino mice by inoculation. Both *C. vitelline* extract (150 mg/kg) and 5-FU (20 mg/kg) reduced the tumor volume by 80% and 69.8%, respectively. Both therapies decreased tumor cell invasion, mitotic activity and increased the formation of apoptotic bodies evaluated using H&E staining and further processed by immunohistochemistry. Notably, mRNA expression of Bax and caspase 3 was decreased and the elevated level of Bcl-2 was detected in solid Ehrlich carcinoma tissue in the treated group [32]. Lichen-derived molecule PHY, isolated from *Pseudocyphellaria granulate*, inhibited the growth of CT26 xenograft in BALB/c mice. The authors observed reduced tumor volume and weight by 55% and 75%, respectively vs control animals after the treatment. In addition, PHY did not reduce the animal body weight [22].

Several lichen-derived acids demonstrated promissing therapeutic efficacy in cancer models in vivo. The study aims of Martins et al. were to perform in vivo evaluations of the anticancer potential of BA isolated from the lichen *Cladia aggregata*. Sarcoma-180 cells were inoculated in the right axillary region of female albino Swiss mice. Results showed that BA did not affect the proliferation of tumor cells, however, it significantly reduced tumor weight. Barbatic acid also demonstrated low toxicity rate in animals. Experimental data in vivo showed a tight cross-connection between the application of secondary metabolite of lichens and antineoplastic events in tumor cells, probably through the activation of the apoptotic cascade leading to cell death [91]. Hypostictic acid *and SA*, *isolated from Pseudoparmelia sphaerospora* and *Parmotrema cetratum* respectively, *showed anti-tumor effects in the murine melanoma model using B16-F10 cell line*. *Authors found that SA reduced tumor volume by 88% and HA by 72% compared to controls*. *Interestingly*, *both acids demonstrated high cancer selectivity associated with low animal toxicity* [79]. Karagoz et al. found the anticancer effect of DA on Ehrlich ascites carcinoma cells (EAC) inoculated into Balb/C mice. Indeed, histopathological and hematological analyses demonstrated that lower concentrations of DA have protective effects in various organs when compared to higher ones [120]. Similarly, in another mice xenograft model, NA reduced both tumor volume and weight of human MDA-MB-231/GFP cells when compared to untreated controls. Western blot analysis of tumor tissue lysates revealed significant inhibition of cell signaling pathway linked with c-Met phosphorylation in treated animals [33]. Table 5 shows an overview of in vivo studies evaluating anticancer effects of extracts or isolated secondary metabolites of lichens.

## 5. Conclusions and Future Directions

Lichens represent a rich source of bioactive molecules that have great potential of their clinical utility in cancer disease. The modern methodologic biomedicinal approaches that facilitate an isolation and characterization of lichen metabolites and consequently define their very complex biological cellular effects, provide suitable conditions for the acceleration of the research which includes the future clinical testing and following use of lichen-derived anticancer drugs in medical practice [121]. In this paper, we have comprehensively reviewed most recent scientific preclinical data of this topic. We have described significant anticancer efficacy of isolated lichen compounds, lichen extracts, or isolated compounds in combination with lichen extracts in cancer cell lines or animal cancer models. Despite above mentioned optimistic scenario about perspective of lichens extracts or isolated lichen molecules as anticancer substances, we did not find any relevant clinical research that provide insight into the anticancer activity of lichen species in humans so far. Therefore, further studies are still needed to determine the potential clinical application and clarify their beneficial effects in cancer patients or risk individuals [9,122]. However, regarding clinical utility of lichens’ secondary metabolites in cancer disease, there are logic limitations that must be addressed to clinical oncologists/researchers. Most of malignant tumors represent very dynamic structures with numerous cell lines that differ in genotypes and phenotypes. There can be observed in such malignancies, a highly variable sensitivity to therapeutics and some of cell lines develop resistance to the treatment, including lichen-derived molecules or extracts [8,14]. The concept using of the drug combination of lichen-derived molecules (or their natural coctails present in extracts) with the conventional therapy to target a wider range of signaling pathways in cancer cells seems to be substantially beneficial compared to using of single anticancer drug (e.g., in the delay of drug resistance, prolonging the progression-free and overall survival in patients or prolonging the cancer latency during long-term chemoprevention in high-risk individuals) [123].

The future preclinical and clinical research focused on isolated lichen metabolites or extracts influencing the carcinogenesis should be directed toward the several important issues: (1) Clarifying the molecular targets and signaling pathways involved in anticancer activity. (2) Determining of an effective (and non-toxic) doses in humans. (3) Assessing a combined effect of several lichen metabolites or extracts targeting several relevant genetic, epigenetic, and immunomodulatory pathways. (4) Investigating epigenetic mechanisms such as modifications of methylation status in gene promoters, posttranslation histone modifications, and expression on miRNAs spectrum. (5) Assessing of the cancer stem cells survival, regarding the relapse, multidrug resistance, or re-sensitizing cancer cells towards standard chemotherapy. (6) Considering the improved bioavailability of lichen metabolites by utilizing, for example, nanoparticles carriers. (7) The detection of new spectrum of lichen-derived bioactive molecules with proved anticancer activities in the form of isolated molecules or extracts, that can potentially provide progressive therapeutic approaches for clinical practice. (8) Chemical structure of most lichen molecules is simple which prompt their easy synthesis. In this regard, many of these synthetic substances may be applied as precursors to fit specific mechanisms of anticancer action, increase stability, and decrease undesirable side effects in the body, that could lead to their improved anticancer activities and provide reasonable clinical use. (10) Later, it could be better understanding of the target mechanisms associated with the individual characteristics with the aim to develop personalized medications from lichens.

## Figures and Tables

**Table 1:** Secondary metabolites of lichens, their origin, chemical structure and activities.

	Origin	Chemical Structure	Activities	References
Usnic acid (UA) 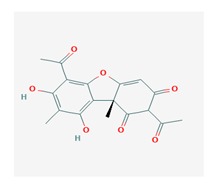	*Usnea diffracta*, *Cladonia arbuscula*,	2,6-diacetyl-7,9-dihydroxy-8,9b-dimethyl-1,3	antimicrobial, antiprotozoal, antiviral, antiproliferate, anti-inflammatory, analgesic, antipyretic	[28,29]
		CAS ID (125-46-2)		
	*Alectoria samentosa*,			
	*Flavocetraria nivalis*,			
	*Alectoria ochroleuca*,			
	*Usnea florida*			
Diffractaic acid (DA) 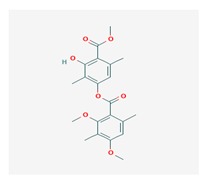	*Usnea longissimi*, *Usnea subcavata*,	4-[(2,4-dimethoxy-3,6-dimethylbenzoyl)oxy]-2-hydroxy-3,6-dimethylbenzoic acid	antioxidant, gastroprotective, analgesic, antiviral,	[38,39,40]
	*Protousnea magellanica*	CAS ID (436-32-8)		
Lobaric acid (LA) 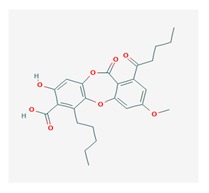	*Stereocaulon alpinum*, *Cladonia sp.*, *Stereocaulon sasakii*	3-hydroxy-9-methoxy-6-oxo-7-(1-oxopentyl)-1-pentyl-2-benzo[b][1,4]benzodioxepincarboxylic acid	antibacterial, antioxidant, antimitotic	[41,42,43]
		CAS ID (522-53-2)		
Atranorin (ATR) 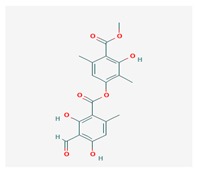	*Parmelia sulcate*, *Parmotrema stuppeum*, *Stereocaulon alpinum*, *Physcia aipolia*	3-hydroxy-4-methoxycarbonyl-2,5-dimethylphenyl	antimicrobial, antiprotozoal, antiviral, antifungal, antioxidant	[44,45]
		CAS ID (479-20-9)		
Vulpinic acid (VA) 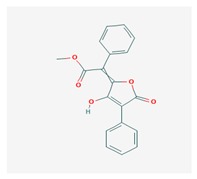	*Letharia vulpina*	methyl (2E)-2-(3-hydroxy-5-oxo-4-phenylfuran-2-ylidene)-2-phenylacetate	antiproliferative, antimicrobial, antiangiogenic,	[46,47]
		CAS ID (73622-57-8)		
Physodic acid (PA) 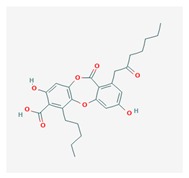	*Hypogymnia physodes*	3,9-dihydroxy-6-oxo-7-(2-oxoheptyl)-1-pentylbenzo[b][1,4]benzodioxepine-2-carboxylic acid	antimicrobial, antioxidant, immunoprotective	[48,49]
		CAS ID (84-24-2)		
Olivetoric acid (OA) 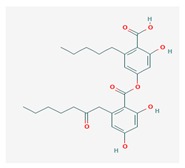	*Pseudevernia furfuracea*	4-[2,4-dihydroxy-6-(2-oxoheptyl)benzoyl]oxy-2-hydroxy-6-pentylbenzoic acid	antimicrobial, antioxidant	[48,50]
		CAS ID (491-72-5)		
Psoromic acid (PSA) 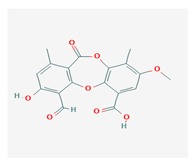	*Usnea camplanata*	10-formyl-9-hydroxy-3-methoxy-4,7-dimethyl-6-oxobenzo[b][1,4]benzodioxepine-1-carboxylic acid	cardioprotective	[51]
		CAS ID (7299-11-8)		
Protocetraric acid (PrA) 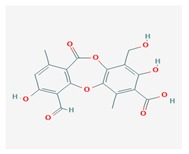	*Parmelia caperata*, *Usnea albopunctata*, *Parmelia saxatilis*, *Parmelia sulcate*	10-formyl-3,9-dihydroxy-4-(hydroxymethyl)-1,7-dimethyl-6-oxobenzo[b][1,4]benzodioxepine-2-carboxylic acid	antimicrobial, immunostimulatory	[52,53]
		CAS ID (489-51-0)		
Norstictic acid (NA) 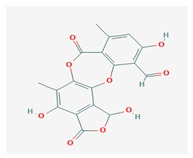	*Toninia candida*	1,3-Dihydro-1,4,10-trihydroxy-5,8-dimethyl-3,7-dioxo-7H-isobenzofuro(4,5-b)(1,4)benzodioxepin-11-carboxaldehyde	antioxidant, antibacterial	[54]
		CAS ID (571-67-5)		
Divaricatic acid (DiA) 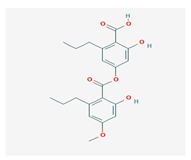	*Evernia mesomorpha*	2-hydroxy-4-[(2-hydroxy-4-methoxy-6-propylbenzoyl)oxy]-6-propylbenzoic acid	antimicrobial, antioxidant	[55]
		CAS ID (491-62-3)		
Perlatolic acid (PeA) 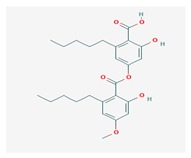	*Cetrelia monachorum*	2-Hydroxy-4-((2-hydroxy-4-methoxy-6-pentylbenzoyl)oxy)-6-pentylbenzoic acid	anti-inflammatory, anti-neurodegenerative	[56,57]
		CAS ID (529-47-5)		
Caperatic acid (CA) 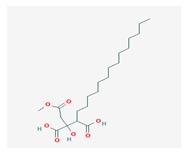	*Platismatia glauca*	2-hydroxy-2-(2-methoxy-2-oxoethyl)-3-tetradecylbutanedioic acid	fungitoxic	[58]
		CAS ID (29227-64-3)		
Lecanoric acid (LeA) 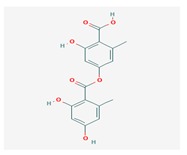	*Usnea subvacata Motyka*, *Parmotrema stuppuem*, *Parmotrema tinctorum and Parmotrema grayana*	4-(2,4-dihydroxy-6-methylbenzoyl)oxy-2-hydroxy-6-methylbenzoic acid	antioxidant	[59]
		CAS ID (480-56-8)		
Barbatic acid (BA) 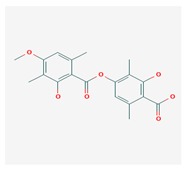	*Usnea longissima*	2-hydroxy-4-(2-hydroxy-4-methoxy-3,6-dimethylbenzoyl)oxy-3,6-dimethylbenzoic acid	antioxidant, antimicrobial	[60,61]
		CAS ID (17636-16-7)		
Barbatolic acid (BrA) 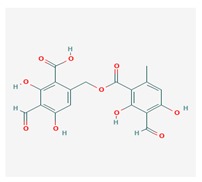	*Bryoria capillaris*	3-formyl-6-[(3-formyl-2,4-dihydroxy-6-methylbenzoyl)oxymethyl]-2,4-dihydroxybenzoic acid	antimicrobial	[35]
		CAS ID (529-50-0)		
Lobastin (LOB) 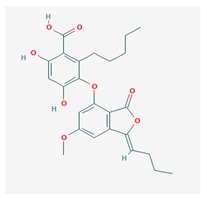	*Stereocaulon alpnum*	3-[[(1Z)-1-butylidene-6-methoxy-3-oxo-2-benzofuran-4-yl]oxy]-4,6-dihydroxy-2-pentylbenzoic acid	antibacterial, antioxidant	[30]
Hypostictic acid (HA) 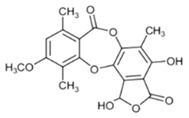	*Pseudoparmelia sphaerospora*	(1,4-dihydroxy-10-methoxy-5,8,11-trimethyl-1H-benzo[e]furo[3′,4′:3,4]benzo[b][1,4]dioxepine-3,7-dione)	antimicrobial	[62]
Salazinic acid (SA) 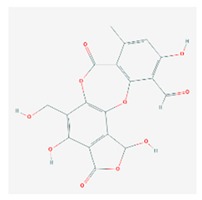	*Parmelia caperata*	5,13,17-trihydroxy-12-(hydroxymethyl)-7-methyl-9,15-dioxo-2,10,16-trioxatetracyclo[9.7.0.03,8.014,18]octadeca-1(11),3(8),4,6,12,14(18)-hexaene-4-carbaldehyde	antibacterial, antifungal, antioxidant, antiviral	[55,63]
		CAS ID (521-39-1)		
Retigeric acid B (RA-B) 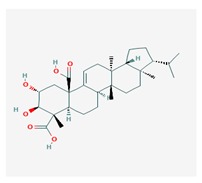	*Lobaria kurokawae*	(3R,3aR,5aR,5bR,7aR,8S,9R,10R,11aR,13aS,13bR)-9,10-dihydroxy-3a,5a,8,13a-tetramethyl-3-propan-2-yl-1,2,3,4,5,5b,6,7,7a,9,10,11,13,13b-tetradecahydrocyclopenta[a]chrysene-8,11a-dicarboxylic acid	antifungal	[64]
		CAS ID (38327-77-4)		
Protolichesterinic acid (PLA) 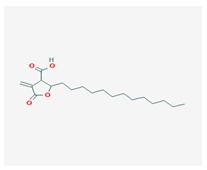	*Cornicularia aculeate*, *Centraria islandica*	4-methylidene-5-oxo-2-tridecyloxolane-3-carboxylic acid	antibacterial, anti-inflammatory	[65]
		CAS ID (1448-96-0)		
Ramalin (RAM) 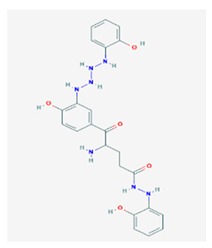	*Ramalina terebrata*	γ-glutamyl-N′-(2-hydroxyphenyl)hydrazide	antioxidant, antibacterial	[66,67]
Physciosporin (PHY) 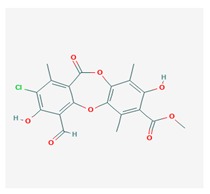	*Pseudocyphellaria granulate*, *Pseudocyphellaria coriacea*	methyl 8-chloro-10-formyl-3,9-dihydroxy-1,4,7-trimethyl-6-oxobenzo[b][1,4]benzodioxepine-2-carboxylate	antiproliferative	[20]
Sekikaic acid (SeA) 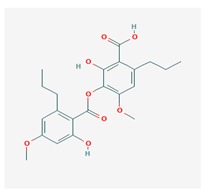	*Cladonia sp.*, *Ramalina roesleri*	2-hydroxy-3-(2-hydroxy-4-methoxy-6-propylbenzoyl)oxy-4-methoxy-6-propylbenzoic acid	antioxidant, antibacterial, antidiabetic	[18]
		CAS ID (607-11-4)		

Abbreviations: *ATR*, atranorin; BA, barbatic acid; BrA, barbatolic acid; CA, caperatic acid; *DA*, diffractaic acid; DiA, divaricatic acid; *GA*, gyrophoric acid; HA, hypostictic acid; *LA*, lobaric acid; LeA, lecanoric acid; Li, lichexanthone; LOB, lobastin; NA, nortictic acid; *OA*, olivetoric acid; *PA*, physodic acid; PeA, perlatolic acid; PLA, protolichesterinicc acid; PrA, protocetraric acid; PSA, psoromic acid; PU, potassium usnate; RA-B, retigeric acid B; RAM, ramalin; SA, salazanic acid; SeA, sekikaic acid; UA, usnic acid; VA, vulpinic acid.

**Table 2:** Anticancer potential of isolated lichen compounds.

Lichen Acid/Species	Cell Lines	Effects	Reference
UA	CaCo2, HepG2, Hep2C, RD, Wehi,	↑ cytotoxicity of CaCo2 (IC_50_ 7.05 μM), HepG2 (IC_50_ 15.4 μM), Hep2C (IC_50_ 21.8 μM), RD (IC_50_ 22.9 μM), Wehi (IC_50_ 15.8 μM)	[29]
		↑ Bax ↓ Bcl-2 ↓ p53	
	HCT116, LS174	↓ SCF-induced proliferation and migration of HCT116 and LS174 (c-KIT+)	[68]
		↑ autophagy of HCT116 (via ↓ mTOR)	
		↓ level of phosphorylated PKC-A, c-KIT of HCT116	
	BGC823, SGC7901	↓ proliferation + G0/G1 and G2/M arrest of BGC823 (IC_50_ 236.55 μM) and SGC7901 (IC_50_618.82 μM)	[69]
		→ apoptosis, ↑ autophagy	
		↑ Bax/Bcl-2 ratio	
		↑ caspase-3, ↑ PARP	
	MCF-7, MDA-MB-231, BT-474	*MDA-MB-231*: *67 UA-responsive miRNAs*	[37]
		*BT-474*: *15 UA-responsive miRNAs*	
		*MCF-7*: *8 UA-responsive miRNAs*	
	HepG2, SNU-449	→ apoptosis and autophagy	[27]
		G0/G1, G2/M arrest	
	A549	→ apoptosis	[28]
		↓ cell number	
		↓ proliferation	
		↓ expression CDK4, CDK6, cyclin D1	
		↑ expression of p21/cip1 protein	
	T47D and MCF-7	Formation of autophagosome (H^+^ shuttling in mitochondria and lysosomes)	[70]
	Capan-2		
	MCF-7	→ apoptosis	[71]
	A549, H460, H1650 and H1975	↓ motility of A549	[19]
		↓ invasion of H1650 and H1975 (↓β-catenin-mediated TOPFLASH and KITENIN-mediated AP-1 activity)	
		↓ expression of CD44, c-myc and Cyclin D1 in all cell lines	
		↓ GTP-Rac1 and RhoA	
Synthetic derivatives of *UA*	MCF-7, PC-3, HeLa	Derivatives 2a, 2b:	[72]
		↓ proliferation of PC-3, MCF-7 (IC_50_ value 3 μM), HeLa (IC_50_ 1 μM)	
		G0/G1 arrest + → apoptosis of MCF-7	
		Activation of cytoplasmic vacuolisation	
		All active derivatives:	
		G0/G1 arrest + ↓ fraction in S and G2/M phase of HeLa	
*PU*	HCT116, DLD1, SW480, HT29, SW620, Caco2, COLO320, CT26	↑ cytotoxicity (lower IC_50_ than UA, except of SW480 and CT26 cells)	[34]
		↓ invasion of Caco2 and HCT116	
		↓ Caco2 motility (↓*CAPN1*, *CDC42*, *CFL1*, *IGF1*, *WASF1*, *WASL*)	
*DA*	U87MG-GBM, PRCC	*LA*: ↑cytotoxicity of *GBM and PRCC* (IC_50_ *of LA*, *DA and UA* 9.08, 122.26, 132.69 mg/L *in PRCC* and 5.77, 35.67 and 41.55 mg/L in U87MG)	[73]
*LA*			
*UA*			
*UA*	HTB-140, DU-145, PC-3	↓ *proliferation*, ↓ *migration*, ↓ *actin organization*	[74]
*ATR*			
UA	HepG2, HUVEC, NS2OY	UA: ↑ cytotoxicity	[75]
VA		↓ proliferation of NS2OY after VA- treatment	
		↑ antiangiogenic effect	
VA	MCF-7, MDA-MB-231, BT-474, SK-BR-3, MCF-12 A	↑ cytotoxicity	[47]
		→ *apoptosis*	
		↑ P53 in SK-BR-3 (vs MCF-12A)	
	CaCo2, HepG2 and Hep2C, RD, Wehi, L929, Vero	*↑ cytotoxicity of* CaCo2 (IC_50_ 13.7 μM), HepG2 (IC_50_ 23.8 μM), Hep2C (IC_50_ 25.3 μM), RD (IC_50_ 34.4 μM), Wehi (IC_50_ 38.6 μM)	[76]
		↓ growth (HepG2, CaCo2, Hep2C, RD, Wehi)	
		↑ Bax and p53 (HepG2, CaCo2, Hep2C, RD, Wehi)	
		↓ Bcl-2 (HepG2, CaCo2, Hep2C, RD, Wehi)	
ATR	4T1, NMuMG	↓ clonogenic potential of 4T1 cells; → caspase-3, PARP cleavage, depletion of Bcl xL (4T1)	[45]
ATR	SK-Hep1, Huh-7, SNU-182	↓ cancer cells growth (concentration, >10 μg/mL	[77]
		↑ necrotic cell death, ↓ migration and invasion (Sk-Hep1, Huh-7)	
		G2/M arrest (SK-Hep1)	
*ATR*	A375	PA (concentration, 6.25–50 μM): ↓ A375, ↑ apoptosis	[17]
*GA*		ATR and GA (high concentrations): ↓ A375	
OA	PRCC	↑ cytotoxicity	[43]
PA	U87MG		
PSA			
ATR	UACC-62	↑ cytotoxicity (PrA, NA, PsA, DiA, PeA against UACC-62)	[78]
PrA	NIH/3T3	↑ selectivity of PrA, NA, PsA, DiA, PeA for UACC-62	
UA	B16-F10	↑ effectivity of NA and DIA against B16-F10	
DA			
Li			
NA			
PeA			
DiA			
PSA			
PA	HCT116, DLD-1, HaCaT	*CA*: *strongest cytotoxic* (*concentration*, *100* μM)	[26]
CA		*CA* (HCT116, DLD-1): ↓ β-catenin regulated expression of Axin2, ↓ migration	
LeA		CA (HCT116): ↓ Axin2	
		CA + PA (HaCat): ↓ MMP7, ↓ survivin	
BA	HeLa, A549, MCF-7, DU-145, HEK293	↓ HeLa (IC_50_ 3.2 μg/mL), A549 (IC_50_ 1.8, 3.2 μg/mL), MCF-7 (IC_50_ 3.2 μg/mL), DU-145 (IC_50_ 9.0 μg/mL)	[38]
		BA (concentration, 1 μM):	
		G0/G1 arrest, ↑ apoptosis, ↑ caspase-3 activity, PARP cleavage, annexin V staining and chromatin condensation (A549)	
BrA	T-47D, HCC1428, HUVECs	↓ *endothelial tube formation*	[35]
		↓ *migration*	
LA	HeLa, HCT116	↓ *viability* (IC_50_ *50* μM)	[30]
LOB		↓ proliferation	
		→ G2/M arrest	
		→ apoptosis (↑ Annexin V-positivity and PARP cleavage,	
		↓ Bcl-2)	
HA, SA	B16-F10, 786-0, HT-29, K562	*HA* (*K562*, *B16-F10*, *786-0*): *↓ proliferation*	[79]
		*SA* (*K*562, HT-29, B16-F10): ↓ *proliferation*	
SA, PA	FemX, LS174	↑ cytotoxicity	[80]
RA-B	PC-3, DU145	→ apoptosis	[81]
		↓ expression of Bcl-2, Bcl-_XL_, cyclin D1, and survivin	
PLA	SK-BR-3, T-47D	↓ proliferation of SK-BR-3	[31]
		↑ expression of fatty acid synthase	
		↓ expression of HER2	
		↓ ERK1/2 and AKT signalling	
	HeLa, SH-SY5Y, K562	PLA with doxorubicin: synergic cytotoxic effect (HeLa)	[82]
	RPMI 8226 and U266	↓ proliferation	[83]
	AsPC-1	→ cell arrest of AsPC-1	
		→ apoptosis (RPMI 8226, U266)	
		↑ cytotoxicity of U266 (IC_50_ 3.5μg/mL), AsPC-1 (IC_50_ 3.5μg/mL), RPMI 8226 (IC_50_1.8 μg/mL)	
*RAM*	HCT116	↓ proliferation (concentration, 50 - 100 μg/mL)	[84]
		↑ G2/M arrest (via ↑ TP53, ↑ p21, ↓ cyclin B1, ↓ CDK1)	
		→ apoptotic cells (concentration, 100 μg/mL)	
		↓ wound healing, invasion, migration	
PHY	Caco2, DLD1, HCT116, SW620, CT26	↑ cytotoxicity of *CT26* (IC_50_ 11.5 μg/mL), SW620 (IC_50_ 12.6 μg/mL), Caco2 (IC_50_ 13.3 μg/mL), HCT116 (IC_50_ 19.8 μg/mL) and DLD1 (IC_50_ 24.9 μg/mL)	[22]
		→ *apoptosis* (*PHY at toxic concentrations*)	
		↓ migration, invasion, colony formation (PHY at non-toxic doses)	
		↓ downstream transcription factors and/or target genes of EM	
		↓ KITENIN, ↓ β-catenin	
		↓ actin-based cell motility	
	A549, H1650, H1975	↓ migration	[85]
		↓ invasion	
antarA	MCF-7, HeLa, A549, NHME	AntarB (concentration, 30 μg/mL): stronger growth inhibition (HeLa, MCF-7) vs doxorubicin (concentration, 10 μg/mL)	[86]
antarB		AntarB and 2′-*O*-methyl DiA: ↓ proliferation of A549 (IC_50_ values of 22.5 and 27.5 μg/mL, respectively)	
SeA		All metabolites: ↓ toxicity against NHME vs cancer cells	
ATR			
DiA			
2′-*O*-methyl DiA			

Explanatory notes: ↑ increase; ↓ decrease; → induction; + plus/and. Abbreviations: 2′-O-methyl DiA, 2′-O-methyl divaricatic acid; antarA, antarvediside A; antarB, antarvediside B; *ATR*, atranorin; BA, barbatic acid; BrA, barbatolic acid; CA, caperatic acid; *DA*, diffractaic acid; DiA, divaricatic acid; *GA*, gyrophoric acid; HA, hypostictic acid; *LA*, lobaric acid; LeA, lecanoric acid; Li, lichexanthone; LOB, lobastin; NA, nortictic acid; *OA*, olivetoric acid; *PA*, physodic acid; PeA, perlatolic acid; PLA, protolichesterinicc acid; PrA, protocetraric acid; PSA, psoromic acid; PU, potassium usnate; RA-B, retigeric acid B; RAM, ramalin; SA, salazanic acid; SeA, sekikaic acid; UA, usnic acid; VA, vulpinic acid.

**Table 3:** An overview of anticancer efficacy of isolated lichen compounds in combination with lichen extracts.

Lichen Acid/Species	Cell Lines	Effects	Reference
UA	HT29, AGS, A549, CWR22Rv-1	↑ selective cytotoxicity (acetone extract and UA)	[87]
Extract of *Flavocetraria cucullata*		↓ tumorigenesis and motility	
		↓ EMT and Akt phosphorylation	
		↑ anticancer activity of extract vs. UA	
Extract of *Toninia candida*,	FemX, LS174	↑ cytotoxicity	[54]
Extract of *Usnea barbata*		→ apoptosis after UA treatment	
NA, UA			
SCE	B16, C6, HaCaT	↑ cytotoxicity of B16 (IC_50_ 31.21 μg/mL) and C6 (IC_50_ 43.40 μg/mL)	[88]
		↑ apoptosis and/or autophagy in B16 and C6	
		Low toxicity against HaCaT	
Acetone extract of *Evernia prunastri*	FemX, LS174	PA: ↑ cytotoxicity LS 174 and FemX	[89]
Acetone extracts of *Pseudoevernia furfuraceae*		↓ FemX and LS174: S and G2/M arrest	
PA			
PA	MCF-7, T47D, MDA-MB-231, MCF-10A	PA: ↑ cytotoxicity of MCF-7 (IC_50_ 72.4 μg/mL), T47D (IC_50_ 75.4 IC_50_ μg/mL), MDA-MB-231 (IC_50_ 93.9 μg/mL)	[24]
Acetone extract of *Hypogymnia physodes*			
Ethanol extract of *Usnea strigosa*	MD-MB-231, MDA-MB-468, MCF-7, T-47D, BT-474, SK-BR-3, MCF-10A	*U. strigosa* extracts: ↑ cytotoxicity of MD-MB-231 (IC_50_ 3.7 μg/mL) MDA-MB-468 (IC_50_ 4.5 μg/mL), MCF-7 IC_50_ 6.4 μg/mL), T-47D (IC_50_ 9.6 μg/mL), BT-474 (IC_50_ 7.9 μg/mL), SK-BR-3 IC_50_ 7.5 μg/mL)	[33]
NA		NA: MD-MB-231(IC_50_ 14.9 μg/mL), MDA-MB-468 (IC_50_ 17.3 μg/mL)	
		↓ proliferation	
		↓ migration of MDA-MB-468	
		↓ invasion of MDA-MB-231	
		↓ c-Met, STAT3, paxillin/Rac-1and FAK phosphorylation in MDA-MB-231	
Acetone extracts of *Melanelia subaurifera and Melanelia fuliginosa*	HeLa, A549, LS174, MRC5	*Melanelia subaurifera extract*: ↑ cytotoxicity of *HeLa* (IC50 9.88 μg/mL) A549 (IC_50_ 31.25 μg/mL), LS174 (IC_50_ 31.64 μg/mL);	[25]
LeA		*Melanelia fuliginosa extract*: *HeLa* (IC_50_ 45.24 μg/mL) A549 (IC_50_ 125.276 μg/mL), LS174 (IC_50_ 142.87 μg/mL);	
2′-O-MA			
*Extract of Cladonia salzmannii*	RAW 264.7, NCI-H292, HEp-2, MCF-7, HL-60	*Ether extract*: cytotoxicity of *HL-60* (IC_50_ 3.59 μg/mL), *HEP-2* (IC_50_ 26.75 μg/mL), *NCI-H292* (IC_50_ 29.91 μg/mL), *RAW-264.7* (IC_50_ 36.54 μg/mL)	[90]
		Acetone extract: cytotoxicity of MCF-7 (7.55 μg/mL) and NCI-H292 (16.60 μg/mL)	
*BA*		Cytotoxicity of HEP-2 (IC_50_ 15.79 μg/mL), MCF-7 (IC_50_ 18.28 μg/mL), RAW-264.7 (IC_50_ 20.79 μg/mL)	
BA	HEp-2, NCI-H292, KB	Cytotoxicity of HEp-2 (IC_50_ 6.25 μg/mL)	[91]
*Acetone extracts of*	A549	↓ *migration* (*concentration*, *10* μg/mL)	[20]
*Everniastrum vexans*			
*ATR*		↑ cytotoxicity (concentration, >5 μg/mL)	
		↓ β-catenin-mediated TOPFLASH activity (via ↓ nuclear import of β-catenin, ↓ c-jun/AP-1)	
		↓ mRNA expression of KITENIN	
		↑ KAI1 mRNA	
		↓ GTP-Cdc42, GTP-RhoA, STAT proteins	
*Extracts of endolichenic fungus EL002332*	AGS, TMK-1, CT26	↑ cytotoxicity (on AGS and CT26)	[92]
		*EL002332 +* docetaxel: synergistic effects (on AGS and TMK-1)	
*myC*		↑ *apoptosis* (*caspase activation*, *Bcl-2 family regulation*)	

Explanatory notes: ↑ increase; ↓ decrease; → induction; + plus/and Abbreviations: 2′-O-MA, 2′-O-methyl anziaic acid; ATR, atranorin; BA, barbatic acid; LeA, lecanoric acid; NA, nortictic acid; PA, physodic acid; SCE, supercritical CO_2_ extract of old´s man beard; UA, usnic acid.

**Table 4:** Anticancer potential of lichen extracts.

Lichen Acid/species	Cell Lines	Effects	Reference
Extract of *Umbilicaria crustulosa*	FemX, LS174	↑ cytotoxicity (all tested extracts)	[93]
Extract of *Umbilicaria cylindrica*			
Extract of *Umbilicaria polyphylla*			
Methanol extract of *Lasallia pustulata*	FemX, LS174	↑ cytotoxicity: FemX (IC_50_ 46.66 μg/mL); LS174 (IC_50_ 71.71 μg/mL)	[94]
Extract of *Parmelia caperata*	FemX, LS174	↑ cytotoxicity (all tested extracts)	[95]
Extract of *Parmelia sulcata*			
Extract of *Parmelia saxatilis*			
Extract of *Parmotrema reticulatum*	MCF-7, A549, WI-38	↑ cytotoxicity	[96]
		→ cell cycle arrest	
Methanol extract of *Cetraria islandica*	FemX, LS174	↑ cytotoxicity: FemX (IC_50_ 22.68 μg/mL); LS174 (IC_50_ 33.74 μg/mL)	[97]
Acetone extract of *Parmelia arseneana*	FemX, LS174, A549, K562	↑ cytotoxicity (IC_50_ 11.61–47.06 μg/mL)	[98]
Water extracts of *Dermatocarpon vellereum*, *Umbilicaria vellea*, *Xanthoria elegans* and *Melanelia disjuncta*	HepG2, RKO	↑ cytotoxicity (all extracts, mainly *L. alphoplaca* and *M. disjuncta*)	[99]
Methanol extracts of *Melanelia disjuncta*, *Lobothallia alphoplaca and Xanthoparmelia stenophylla*			
Methanol extracts of *Parmelia sulcata* Taylor and *Usnea filipendula* Stirt	A549, PC-3, Hep3B	↑ cytotoxicity (IC_50_ 32.9–98.5 μg/mL)	[100]
	Rat glioma C6		
		→ genotoxicity	
		→ apoptotosis	
Methanol extract of *Hypogymnia physodes*	MCF-7, MDA-MB-231	↑ anticancer and/or apoptosis-inducing (low concentration) effect	[101]
		↑ genotoxicity (high concentration)	
Methanol extracts of *Cladonia rangiformis* and *Cladonia convolute*	MCF-7	→ apoptosis	[102]
		↓ proliferation	
		↑ cytoxicity	
Acetone extract of *Xanthoria parietina*	MCF-7, MDA-MB-231	↓ proliferation	[103]
		↓ cell cycle	
		↑ apoptosis	
Metanol extract of *Parmelia sulcata*	MCF-7, MDA-MB-231	↑ cytotoxicity MCF-7 (IC_50_39.1 μg/mL); MDA-MB-231 (IC_50_ 16.5 μg/mL)	[104]
		→ apoptosis	
Extracts of *Xanthoparmelia chlorochroa* and *Tuckermannopsis ciliaris*	Human Burkitt’s lymphoma (Raji)	→ apoptosis	[105]
		→ cell arrest	
		↑ p53 expression	
Acetone extracts of	AGS, A549, MDCK	*P. gardneri*: ↑ cytotoxicity of AGS (IC_50_ 39.1 μg/mL), A549 (IC_50_ 20.24 μg/mL), MDCK (IC_50_ 66.35 μg/mL); *Canoparmelia aptata*: AGS (IC_50_ 167.9 μg/mL), A549 (IC_50_ 200 μg/mL)	[106]
*Parmotrema gardneri*, *Pannaria sp.*, and *Canoparmelia aptata*			
Extract of *Cladonia rangiformis*	MCF-7	↑ cytotoxicity	[107]
Extract of *Cladonia convoluta*			
*Caloplaca pusilla* (*on G-LBM medium*)	HeLa, MCF-7, PC-3	↓ viability of MCF-7 (IC_50_ 7.29 μg/mL), PC-3 (IC_50_ 7.96 μg/mL), HeLa (IC_50_ 6.57 μg/mL)	[108]
		→ apoptosis	
*Xanthoria parietina* (*on PDA and G-LBM*)		↓ cancer cell viability of MCF-7, HeLa (IC_50_ about 8 μg/mL)	
*Methanol extract of Cladonia pocillumon*	MCF-7	→ *apoptosis* (*concentration-dependent*)	[109]
*Acetone extract of Pleurosticta acetabulum*	HT-29	↑ cytotoxicity (IC_50_ after 48 h, 6 μg/mL)	[110]
(*cytochalasin E*)		↓ proliferation	
		→ apoptosis	
*Polysaccharide from Umbilicaria esculenta*	A875, A375, HUVEC	↑ cytotoxicity of A875 and A375	[111]
		↑ Annexin-V positive and TUNEL positive A875	
		→ apoptosis of A875 (ROS generation followed by ↑ caspase-3 and -9)	
Acetone extracts of *Cladonia furcata* and *Cladonia foliacea*	HeLa	*Extract of C. foliacea*: ↑ cytotoxicity of *A549* (IC_50_ 13.58 μg/mL), *LS174* (IC_50_ 28.98 μg/mL)	[112]
	Human lung carcinoma A549	Extract of *C. furcata*: ↑ cytotoxicity of HeLa (IC_50_ 11.69 μg/mL)	
	Human colon carcinoma LS174		
Extract of *Candelariella vitelline*	Caco-2	↓ proliferation (Ki-67)	[32]
		→ apoptosis, ↑ necrosis (Caco-2, IC_50_ 125 μg/mL)	
		↓ Bcl-2	
		↑ Bax, ↑CASP3 protein level	
		↑ Bax/Bcl-2 ratio	
Methanol extract of *Usnea intermedia*	A549, H1299 MCF7, MDA-MB-231	↓ proliferation of H1299 (IC_50_ 10.2 μg/mL) and MDA-MB-231 (IC_50_ 3.0 μg/mL)	[113]
		→ apoptosis (phophatidylserine translocation, ↑ caspase 3/7 activity, loss of mitochondrial membrane potential, formation of pyknotic nuclei)	

*Nemania serpens* and *Nemania aenea var. aureolatum* (isolates of endolichenic fungi associated with the lichen *Nephroma laevigatum*)	HT-29, HCT116, PC-3 and DU145	↑ anticancer efficacy (IC_50_ 13–39 μg/mL)	[114]
		→ apoptosis (activated caspase 3, 8, PARP cleavage, chromatin fragmentation)	

*Physcia* cf. *Milegrana*	HeLa, Vero	↑ cytotoxicity of HeLa (IC_50_ 137 μg/mL)	[115]

Explanatory notes: ↑ increase; ↓ decrease; → induction.

**Table 5:** In vivo studies dealing with anticancer effects of extracts or isolated secondary metabolites of lichens.

Lichen Metabolites/EXTRACS	Model	Effects	References
UA	Bcap-37 cells inoculated s.c. into C57BL/6 female nude mice; chick embryo chorioallantoic membrane assay; mouse corneal angiogenesis model	↓ angiogenesis and VEGFR2 mediated ERK1/2 and AKT signaling; ↓ Bcap-37 cells growth; ↓ proliferation, migration, and tube formation and ↑ apoptosis of HUVEC cells	[116]
	Human breast cancer MCF-7 cells inoculated s.c. into Balb/c nude mouse	↓ tumor growth in dose dependent manner; any toxic effect in animals	[71]
	H22 cells inoculated into male Kunming mice	↓ toxicity of bleomycin therapy; ↑ efficacy of combined therapy vs bleomycine alone-arrested tumor cells in G0/G1; ↑ caspase-3 and -8; ↓ levels of MDA, hydroxyproline, TNF-α, IL-1β, IL-6 and TGF-β1 and ↑ levels of SOD; ↓ *p*-Smad2/3; ↑ Smad7 proteins	[36]
UA and its benzylidene analogue	Human breast cancer MDA-MB-231 and MCF-7 cells inoculated into athymic nude mice	↑ anticancer activity on both xenograft models; ↑ autophagy; ↓ mTOR signaling	[117]
UA	Human gastric carcinoma BGC823 cells inoculated s.c. into the flank of female BALB/C nude mice	↓ tumor volume and weight; ↑ tumor ratio of Bax/Bcl-2 compared to 5-FU	[69]
Flavocetraria cucullata extract, UA (*F. cucullata*), LiA (*F. cucullata*)	Human lung cancer A549 cells injected s.c. into the flank region of Balb/c nude mouse	tumor free survival number: *F. cucullata* group ˃ UA group ˃ LiA group.	[87]
UA and PU	Mouse colorectal cancer CT26-Fluc cells inoculated by intrasplenic injection of male BALB/c mice	↓ tumor growth in orthotopic liver metastasis model; ↓ levels of EMT; PU without hepatotoxic effect in liver metastasis model	[34]
Ethyl acetate extract of *Usnea longissimi*	Gastric and esophageal adenocarcinomas of Albino Wistar male rats induced by oral N-methyl-N-nitro-N-nitrosoguanidin administration	↓ tumor formation; extract concentrations of 50 and 100 mg/kg demonstrated selectivity to cancer tissue and low toxicity profile in animals	[118]
ATR	Mouse breast carcinoma 4T1 cells inoculated s.c. into BALB/c mice	↑ survival time of tumor-bearing animals; ↓ tumor volume; ↑ apoptosis; ↓ oxidative stress in livers of tumor-bearing mice	[45]
	Mouse Lewis lung carcinoma cells inoculated s.c. into the flanks of C57BL/6 mice	↓ tumor volume and weight; ↓ Ki-67; ↓ KITENIN, CD44, STAT, and cyclin-D1	[20]
Extract of *Rocella montagnei*	Dalton’s lymphoma ascites cells inoculated into Albino Wistar rats and consequent cancer fluid aspiration from rat peritoneal cavity injected into new animals	↓ tumor volume; effect comparable to Vincristine	[119]
Endolichenic fungus EL002332 (*Endocarpon pusillum*)	Mouse colorectal cancer CT26 cells inoculated s.c. into BALB/c syngeneic mice; TMK1 cells injected into the abdominal cavity of BALB/c mice (intraperitoneal xenografts)	↓ tumor score and tumor volume in skin and intraperitoneal tumor-bearing animals	[92]
Extract of *Candelariella vitelline*	Ehrlich ascites carcinoma cells were injected i.p. and consequently transferred every 5 days into new female Swiss albino mice	↓ tumor volume; ↓ tumor cell invasion and mitotic activity; ↑ formation of apoptotic bodies; ↑ ratio of Bax/Bcl-2 on both mRNA and protein levels	[32]
Physciosporin (*Pseudocyphellaria granulata*)	Mouse colorectal cancer cells CT26 implanted s.c. into male BALB/c mice	↓ tumor volume and weight; without changes in body weight of animals	[22]
BA (*Cladia aggregate*)	Sarcoma-180 cells inoculated in the right axillary region of female albino Swiss mice	↓ tumor weight; ↑ apoptosis (supposed mechanism)	[91]
HA (*Pseudoparmelia sphaerospora*)	Murine melanoma B16-F10 inoculated s.c. into male BALB/c mice	↓ tumor volume in both acids; high cancer selectivity and low toxicity in both acids	[79]
SA (*Parmotrema cetratum*)			
DA (*Usnea longissima*)	Ehrlich ascites carcinoma (EAC) cells inoculated i.p. to Balb/C male mice	anticancer effect on EAC cells; protective activity on different mouse organs	[120]
NA (*Usnea strigosa*)	Human breast cancer MDA-MB-231/GFP cells inoculated into female nude mice	↓ tumor volume and weight; ↓ c-Met phosphorylation	[33]

Explanatory notes: ↑ increase; ↓ decrease; → induction; *Abbreviations*: ATR, atranorin; BA, barbatic acid; *DA*, diffractaic acid; EMT, epithelial–mesenchymal transition; HA, hypostictic acid; LiA, lichesterinic acid; NA, nortictic acid; PU, potassium usnate; SA, salazanic acid; UA, usnic acid.
